# Diagnosis of Lung Cancer by ATR-FTIR Spectroscopy and Chemometrics

**DOI:** 10.3389/fonc.2021.753791

**Published:** 2021-09-30

**Authors:** Xien Yang, Quanhong Ou, Kai Qian, Jianru Yang, Zhixun Bai, Weiye Yang, Youming Shi, Gang Liu

**Affiliations:** ^1^ School of Physics and Electronic Information, Yunnan Normal University, Kunming, China; ^2^ Department of Thoracic Surgery, The First People’s Hospital of Yunnan Province, Kunming, China; ^3^ Department of Clinical Laboratory, Affiliated Hospital of Zunyi Medical University, Zunyi, China; ^4^ Department of Internal Medicine, The Second Affiliated Hospital of Zunyi Medical University, Zunyi, China; ^5^ School of Physics and Electronic Engineering, Qujing Normal University, Qujing, China

**Keywords:** lung cancer, serum, ATR-FTIR spectroscopy, Beer-Lambert law, chemometrics

## Abstract

Lung cancer is the leading cause of cancer-related death in the world. Early diagnosis has great significance for the survival of patients with lung cancer. In this paper, attenuated total reflectance Fourier transform infrared (ATR-FTIR) spectroscopy combined with chemometrics was used to study the serum samples from patients with lung cancer and healthy people. The results of spectral band area comparison showed that the concentrations of protein, lipid and nucleic acids molecules in serum of patients with lung cancer were increased compared with those in healthy people. The original spectra were preprocessed to improve the accuracy of principal component regression (PCR) and partial least squares-discriminant analysis (PLS-DA) models. PLS-DA results for first derivative spectral data in nucleic acids (1250-1000cm^-1^) band showed 80% sensitivity, 91.89% specificity and 87.10% accuracy with high ${\text{R}}_{\text{c}}^{2}$ of 0.8949 and ${\text{R}}_{\text{v}}^{2}$ of 0.8153, low RMSEC of 0.3136 and RMSEV of 0.4180. It is shown that ATR-FTIR spectroscopy combined with chemometrics might be developed as a simple method for clinical screening and diagnosis of lung cancer.

## Introduction

Lung cancer is the leading cause of cancer-related death in the world. It causes more than 2.2 million new cancer cases and 1.8 million deaths, accounting for 18% of all cancer deaths in 2020 (1). The cause of lung cancer is largely attributed to smoking and genetic inheritance (2, 3). Lung cancer lacks early diagnostic biomarkers. Most patients with lung cancer are already in advanced stage when diagnosed. The treatment effect of patients with advanced lung cancer is poor, and the survival rate is still expected to be less than 15% (4, 5). Therefore, early diagnosis has great significance for the survival of patients with lung cancer.

The traditional clinical diagnosis method is based on the histological examination of tumor tissue samples, but it is invasive and difficult to repeatedly biopsy for dynamic monitoring (6). Common screening methods, such as chest X-rays (CXR), magnetic resonance imaging (MRI) and low-dose computed tomography (LDCT), have some disadvantages. CXR examination cannot fully show early lung lesions with high false negatives. MRI has low sensitivity and cannot be used in patients with particular metal-based implants, pacemakers, and those suffering from acute claustrophobia (7). LDCT has high rates of false-positive results and adverse effects caused by exposure to hazardous radiation (4, 8–10). The existing light-induced theranostic platforms also have several limitations such as tissue absorption and limited imaging (11). Therefore, there is a need for a sample diagnostic method for the diagnosis of lung cancer.

In recent years, vibrational spectroscopic techniques such as Fourier transform infrared (FTIR) spectroscopy and Raman spectroscopy have been widely used in biological samples due to their low cost and small sample consumption (12). Unfortunately, Raman spectroscopy has some limitations because of its strong fluorescence background and weak signal. However, these limitations are not associated with infrared spectroscopy (13). There have been reported that infrared spectroscopy was used to analyze the lung tissue. Bangaoil et al. classified malignant and benign lung tissue sections using infrared spectroscopy combined with principal component analysis (PCA) and hierarchical cluster analysis (HCA). The finally analysis results were consistent with histopathological conclusions (14). Kaznowska et al. studied the tissue samples from healthy people and patients with lung cancer using infrared spectroscopy, and found the corresponding wavenumber changes of the functional groups in lipids, carbohydrates, proteins, DNA and phospholipids (15). However, the tissue samples in these studies still need to be collected from surgery, which is highly invasive. Wang et al. studied the FTIR spectra of serum samples by drying the serum on BaF2 window under vacuum, and found that there were differences in the protein secondary structure of serum between the patients with lung cancer and healthy people (16). However, there is still a lot of work to be done in the practical application of infrared spectroscopy in the clinical diagnosis of lung cancer.

In this paper, ATR-FTIR spectroscopy combined with chemometrics was used to study the serum samples from patients with lung cancer and healthy people in order to explore a simple diagnostic method for lung cancer and lay the foundation for the clinical application of infrared spectroscopy in the diagnosis of lung cancer in the future.

## Materials and Methods

### Samples Preparation

Serum samples were provided by The First People’s Hospital of Yunnan Province. All subjects had given informed consent to be included before they participated in the study. The study was conducted in accordance with the Declaration of Helsinki, and the protocol was approved by the Ethics Committee of Yunnan Normal University (Number: ynnuethic2021-13). Serum samples from 92 patients with lung cancer and 155 samples from healthy people were collected. The information of patients with lung cancer and healthy people was listed in **Table 1**. 50μl serum samples was placed on a glass slide and dried in a vacuum oven at room temperature (25°C) for 40 minutes. The purpose of vacuum pumping is to accelerate the drying speed. Drying in the vacuum drying oven can ensure that the sample will not be polluted and oxidized, and the organic substance will not be destroyed within 40 minutes. Then the serum was removed from the slide for measuring ATR-FTIR spectrum. Before sampling, the glass slides were soaked with aqua regia for 1 hour, washed with water and then soaked in acetone for 1 hour, and finally washed with ultrapure water and dried for use.

**Table 1:** The information of patients with lung cancer and healthy people.

	Mean age ± SD	Sex	
		Male (n)	Female (n)
Patients with lung cancer	56 ± 9	59	33
Healthy people	42 ± 12	96	59

### ATR-FTIR Spectroscopy

ATR-FTIR spectra were measured in the range of 4000-600cm^-1^ by a Frontier spectrometer (Perkin Elmer, UK), coupled with an ATR accessory and a deuterated triglycine sulfate (DTGS) detector. Each spectrum was an accumulation of 32 scans at a resolution of 4cm^-1^. The dried serum sample was transferred to the crystal plate, and then pressed with pressure tip to ensure the best contact with the crystal surface. The air background spectrum was recorded before each sample scan and automatically deducted when the sample was tested. After each measurement, the crystal surface was cleaned with ethanol and ultrapure water, and then dried with a dust-free paper. Three IR spectra were collected for each serum sample and the resulting spectra were averaged using OMNIC 8.2 software (Thermo Scientific).

### Spectral Data Preprocessing

The influence of noise and irrelevant information can be eliminated by proper preprocessing of the original spectra. This increases the accuracy of the analytical model and improves the signal-to-noise ratio (17). Baseline correction (BL) is a necessary processing method in infrared spectroscopy, which is helpful for further qualitative or quantitative analysis (18). Savitzky-Golay (SG) smoothing is adopted to increase the spectral quality by eliminating random noise. Derivative processing can eliminate background interference and spectral overlap, and minimize baseline drift caused by the differences in optical setups (19). Multiplicative scatter correction (MSC) is aimed to effectively eliminate the influence of scattering and improve the spectral information to obtain a relatively ideal spectrum (20). Standard normal variate (SNV) is used to reduce baseline shifting or tilt due to scattering and the change of light distance (21). It subtracts the average intensity from the spectral intensity to achieve offset correction, and then divides the standard deviation to reduce the multiplicative effect (22).

### Spectral Band Area Analysis

The spectral band area was measured using OriginPro 9.1 software (OriginLab Corporation, Northampton, MA). The obtained results were presented as mean ± SEM (standard mean error). For statistics, independent sample t test was processed using SPSS 19 software (SPSS, Inc., Chicago, IL) and GraphPad Prim 9.0 (GraphPad Software Inc., CA, USA). The statistical significance was signified as less than or equal to p < 0.05*, p < 0.01**, p < 0.001***, and p < 0.0001****.

### Chemometrics Analysis

Principal component regression (PCR) and partial least squares-discriminant analysis (PLS-DA) were performed to analyze the spectral data using Unscrambler X 10.4 software (Camo Software AS, Oslo, Norway). PCR is a regression analysis based on PCA (23). It decomposes the X matrix by PCA, and then takes the transformed new variables as predictive variables for multiple linear regression (MLR) (24). PLS-DA is a linear supervised classification technique combining partial least squares (PLS) regression with linear discriminant analysis (LDA) (25). It can find variables and directions from the multivariate space to distinguish the categories in the calibration set (26).

After preprocessing the spectral data, they were randomly divided into calibration set (69 serum samples from patients with lung cancer and 116 serum samples from healthy people) and validation set (23 serum samples from patients with lung cancer and 39 serum samples from healthy people) according to the ratio of 3:1 for model work. The performance of the regression model was evaluated by calculating the square of the correlation coefficient (R^2^) and the root mean square error (RMSE) (27). The sensitivity, specificity and accuracy were used to evaluate the judgment ability of the diagnostic model. The corresponding formula is as follows:


(1) $$\text{Sensitivity}=\frac{\text{TP}}{\text{TP}+\text{FN}}\times 100\%$$

(2) $$\text{Specificity}=\frac{\text{TN}}{\text{TN}+\text{FP}}\times 100\%$$

(3) $$\text{Accuracy}=\frac{\text{TP}+\text{TN}}{\text{TP}+\text{TN}+\text{FP}+\text{FN}}\times 100\%$$

## Results and Discussion

### ATR-FTIR Spectra of Serum

**Figure 1:**
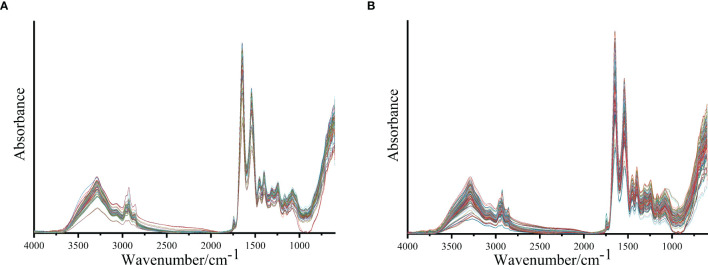
The IR spectra after baseline correction and SG smoothing (9-point) of serum samples from patients with lung cancer **(A)** and healthy people **(B)**.

**Figure 2:**
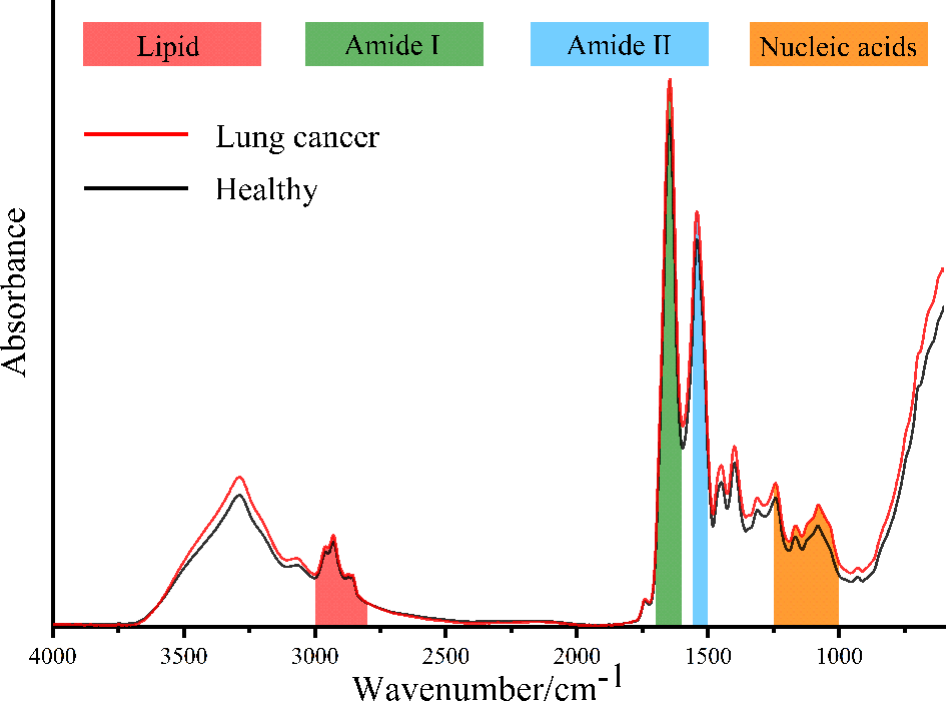
The average IR spectra of the serum from patients with lung cancer and healthy people.

**Figure 1** shows the IR spectra after baseline correction and SG smoothing (9-point) of serum samples from patients with lung cancer and healthy people. The average IR spectra of them are shown in **Figure 2**. It can be seen that the main components of serum are protein, lipid and nucleic acids. The amide I protein (1700-1600cm^−1^) band mainly originated from the α-helix structure at 1646cm^-1^ (28). The amide II protein (1560-1500cm^−1^) band mainly originated from the N-H functional group at 1542cm^-1^ (29). The peak at 1740cm^−1^ was attributed to the C=O stretching vibration from ester carbonyl in triglycerides (25). The spectral band of 3000-2800cm^−1^ was mainly correlated with the lipid-related C-H asymmetric stretching vibration of CH_3_ at 2959cm^-1^ and CH_2_ at 2930cm^-1^ (30, 31). The spectral band of 1250-1000cm^−1^ was correlated with the P=O asymmetric stretching vibration at 1243cm^-1^ and symmetric stretching vibration at 1079cm^-1^ of $\text{P}{\text{O}}_{2}^{-}$ in nucleic acids (32). It could be observed that the absorbance of average IR spectrum in serum from patients with lung cancer was significantly increased at nucleic acids band compared with healthy people. However, there were no significant differences in amide I, amide II and lipid bands in average IR spectra.

### Comparison of Spectral Band Area

**Figure 3:**
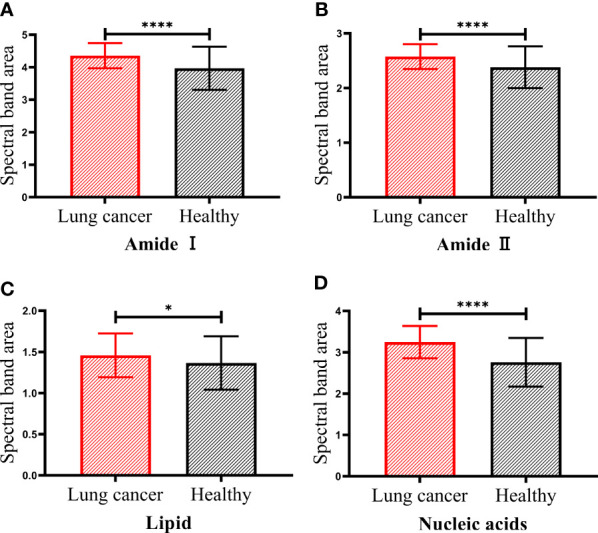
The spectral band area of patients with lung cancer and healthy people. Amide I protein (1700-1600cm^−1^) band **(A)**, Amide II protein (1560-1500cm^−1^) band **(B)**, lipid (3000-2800cm^−1^) band **(C)** and nucleic acids (1250-1000cm^−1^) band **(D)**. The statistical significance was signified as less than or equal to p < 0.05* and p < 0.0001****.

To further analyze the differences between serum of patients with lung cancer and healthy people in these four bands, we showed the statistical analysis results of the spectral band area of serum samples in **Figure 3**. It can be observed that the spectral band area of patients with lung cancer was significantly increased in amide I, amide II and nucleic acids bands compared with healthy people (p < 0.0001). Although the increased area in lipid band was not significant compared with the other three bands, there was still a statistical difference between the two groups of serum samples (p < 0.05). According to Beer-Lambert law, the increase of the absorbance of the spectral band indicates the increase of the corresponding functional group concentration (31). Therefore, the concentrations of protein, lipid and nucleic acids molecules in serum of patients with lung cancer were increased compared with those in healthy people. This may be due to the aerobic glycolysis in cancer cells that produces a large number of biosynthetic intermediates such as lipid, protein and nucleotide, which are used for the growth and proliferation of cancer cells (33).

### Chemometrics Analysis

In order to evaluate the classification effect of these four bands on serum of patients with lung cancer and healthy people, PCR and PLS-DA were performed on the spectral data after preprocessing using full cross-validation. A model with a low value of RMSE and a high value of R^2^ closer to 1 is considered to be a good model (24, 34).

**Tables 2**, **3** show the calibration and validation results of PCR and PLS-DA. Although good classification accuracy appeared under the PCR model, the values of R^2^ and RMSE were lower than those of PLS-DA. The PLS-DA model for first derivative spectral data in nucleic acids band (1250-1000cm^-1^) showed the best calibration model with ${\text{R}}_{\text{c}}^{2}$ of 0.8949 and RMSEC of 0.3136. The values of ${\text{R}}_{\text{v}}^{2}$ and RMSEV in its corresponding validation model were 0.8153 and 0.4180, respectively. The results of this model showed 80% sensitivity, 91.89% specificity and 87.70% accuracy.

**Table 2:** Calibration and validation results of PCR for the spectral data after preprocessing.

Spectral band	Preprocessing	${\text{R}}_{\text{c}}^{2}$	RMSEC	${\text{R}}_{\text{v}}^{2}$	RMSEV	Sensitivity	Specificity	Accuracy
3000-2800cm^-1^	BL+SG 1D 2D 2D+MSC 2D+SNV	0.2096 0.2142 0.2584 0.3773 0.4332	0.8599 0.8574 0.8329 0.7632 0.7282	0.1607 0.1675 0.1969 0.3292 0.3819	0.8909 0.8873 0.8715 0.7965 0.7645	44% 88.46% 81.82% 41.84% 62.96%	67.57% 100% 87.50% 75.61% 82.86%	58.07% 95.16% 85.48% 70.97% 74.19%
1700-1600cm^-1^	BL+SG 1D 2D 2D+MSC 2D+SNV	0.6979 0.6033 0.4650 0.4802 0.4845	0.5316 0.6092 0.7074 0.6973 0.6945	0.6790 0.5768 0.4437 0.4547 0.4587	0.5510 0.6326 0.7253 0.7181 0.7155	95.83% 80% 74.07% 70% 72.73%	100% 83.33% 91.43% 78.57% 82.5%	98.39% 82.26% 83.87% 75.81% 79.03%
1560-1500cm^-1^	BL+SG 1D 2D 2D+MSC 2D+SNV	0.5930 0.6028 0.2252 0.4719 0.4091	0.6171 0.6095 0.8513 0.7029 0.7436	0.5773 0.5729 0.1680 0.4373 0.3684	0.6323 0.6355 0.8870 0.7295 0.7728	100% 89.47% 67.86% 73.08% 72.22%	100% 86.05% 88.24% 88.89% 77.27%	100% 87.10% 79.03% 82.26% 75.81%
1250-1000cm^-1^	BL+SG 1D 2D 2D+MSC 2D+SNV	0.5743 0.5954 0.6895 0.5426 0.5235	0.6311 0.6152 0.5390 0.6541 0.6676	0.5619 0.5612 0.6643 0.5134 0.5195	0.6437 0.6442 0.5635 0.6784 0.6741	100% 95.24% 100% 100% 100%	86.67% 92.68% 86.67% 81.25% 82.93%	90.32% 93.55% 90.32% 85.48% 80.65%

${\text{R}}_{\text{c}}^{2}$ , The square of the correlation coefficient in the calibration set; RMSEC, The root mean square error of calibration set; ${\text{R}}_{\text{v}}^{2}$ , The square of the correlation coefficient in the validation set; RMSEV, The root mean square error of validation set; 1D, First derivative spectra; 2D, Second derivative spectra.

**Table 3:** Calibration and validation results of PLS-DA for the spectral data after preprocessing.

Spectral band	Preprocessing	${\text{R}}_{\text{c}}^{2}$	RMSEC	${\text{R}}_{\text{v}}^{2}$	RMSEV	Sensitivity	Specificity	Accuracy
3000-2800cm^-1^	BL+SG 1D 2D 2D+MSC 2D+SNV	0.4733 0.6879 0.5830 0.6014 0.5866	0.7019 0.5404 0.6246 0.6106 0.6219	0.4185 0.5220 0.4262 0.4584 0.4965	0.7416 0.6723 0.7367 0.7157 0.6900	84.21% 80% 68.97% 62.96% 59.38%	83.72% 91.89% 90.91% 82.86% 86.67%	83.87% 87.10% 80.65% 74.19% 72.58%
1700-1600cm^-1^	BL+SG 1D 2D 2D+MSC 2D+SNV	0.6699 0.7027 0.6411 0.6184 0.6077	0.5557 0.5274 0.5794 0.5975 0.6058	0.6657 0.6320 0.5742 0.5459 0.5417	0.5623 0.5899 0.6346 0.6553 0.6583	85.19% 73.91% 61.11% 71.43% 76%	100% 84.62% 96.15% 80.49% 89.190%	93.55% 80.65% 75.81% 77.42% 83.87%
1560-1500cm^-1^	BL+SG 1D 2D 2D+MSC 2D+SNV	0.6526 0.7070 0.6331 0.5892 0.5982	0.5700 0.5235 0.5858 0.6199 0.6131	0.6210 0.6641 0.5857 0.5118 0.5345	0.5987 0.5636 0.6259 0.6794 0.6635	65.71% 84.62% 85.19% 100% 95.46%	100% 97.22% 100% 97.5% 95%	80.65% 91.94% 93.55% 98.39% 95.16%
1250-1000cm^-1^	BL+SG **1D** 2D 2D+MSC 2D+SNV	0.6930 **0.8949** 0.8516 0.8105 0.7916	0.5359 **0.3136** 0.3726 0.4210 0.4415	0.6529 **0.8153** 0.7741 0.6965 0.6827	0.5729 **0.4180** 0.4622 0.5358 0.5478	91.30% **80%** 100% 88% 94.74%	94.87% **91.89%** 97.5% 97.30% 88.37%	93.55% **87.10%** 98.39% 93.55% 90.32%

${\text{R}}_{\text{c}}^{2}$ , The square of the correlation coefficient in the calibration set; RMSEC, The root mean square error of calibration set; ${\text{R}}_{\text{v}}^{2}$ , The square of the correlation coefficient in the validation set; RMSEV, The root mean square error of validation set; 1D, First derivative spectra; 2D, Second derivative spectra. The bold values in this table are the best preprocessing conditions and results of the model in this article. The purpose of bold is to facilitate the reader's reading, and it is not necessary to deliberately annotate it.

**Figure 4:**
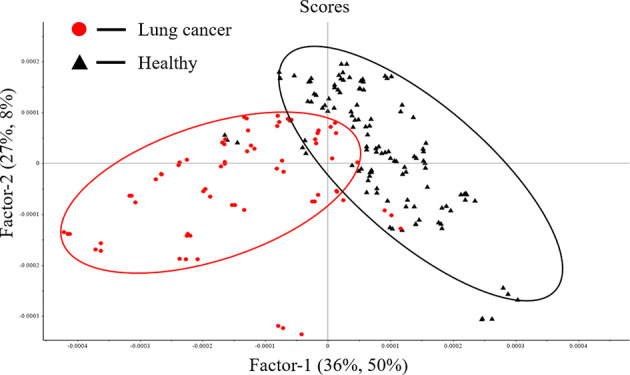
Score plot of Factor-1 and Factor-2.

**Figure 4** shows the score plot of Factor-1 and Factor-2 using PLS-DA model for first derivative spectral data in nucleic acids (1250-1000cm^−1^) band. The first two factors indicate that 65% (X1 36%, X2 27%) of the X variance, explains 58% (Y1 50%, Y2 8%) of the sample classification level. It can be seen that serum samples are distributed into two clusters along the Factor-1. The red cluster is mainly composed of serum samples from patients with lung cancer, and the black cluster is mainly composed of serum samples from healthy people. In this model, 80% of patients with lung cancer were correctly identified, 91.89% of healthy people were correctly separated, and the total accuracy rate was 87.10%.

**Figure 5:**
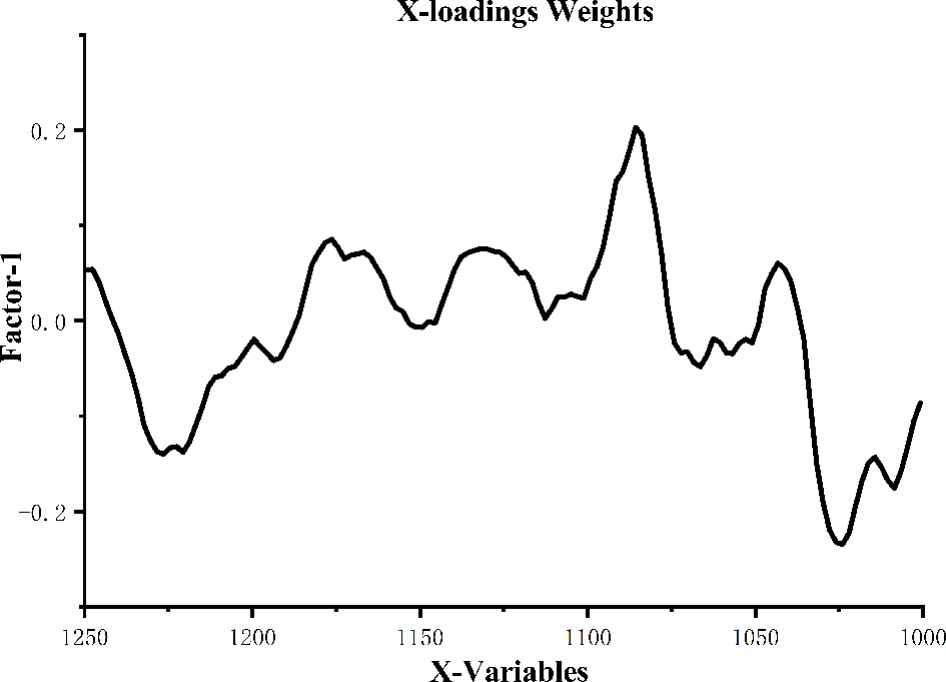
Loading plot of Factor-1.

**Figure 5** shows the loading plot of Factor-1 for identifying the peaks with high weights in classifying samples. There are positively weighted peaks around 1176cm^-1^, 1130cm^-1^, 1085cm^-1^ and 1043cm^-1^, of which 1176cm^-1^ was related to the vibration band of sugar-phosphate, 1130cm^-1^ was assigned to the C=O stretching vibration of ribose in RNA (32), 1085cm^-1^ was ascribed to the symmetric phosphate vibrations (35), 1043cm^-1^ was attributed to the stretching vibration and bending vibration of C-O in carbohydrates (14). Two positively weighted peaks at 1226cm^-1^ and 1026cm^-1^, of which 1226cm^-1^ was due to the asymmetric stretching vibration of $\text{P}{\text{O}}_{2}^{-}$ in nucleic acids (36), 1026cm^-1^ was related to the stretching vibration of C-O and bending vibration of C-H in aromatic amino acids (37). Therefore, the loading plot of Factor-1 showed that $\text{P}{\text{O}}_{2}^{-}$ in nucleic acids play a key role in distinguishing the serum patients with lung cancer from that of healthy people. This may be due to DNA damage caused by oxidative chemical mutagenic aberrations in serum of patients with lung cancer (38).

## Conclusions

ATR-FTIR spectroscopy combined with chemometrics was used to study the serum of patients with lung cancer and healthy people. The results of spectral band area comparison showed that the concentrations of protein, lipid and nucleic acids molecules in serum of patients with lung cancer were increased compared with those in healthy people. PCR and PLS-DA were performed on the spectral data after different preprocessing. PLS-DA model for first derivative spectral data in nucleic acids (1250-1000cm^-1^) band is the best model with high ${\text{R}}_{\text{c}}^{2}$ of 0.8949 and ${\text{R}}_{\text{v}}^{2}$ of 0.8153, low RMSEC of 0.3136 and RMSEV of 0.4180. The corresponding PLS-DA results showed 80% sensitivity, 91.89% specificity and 87.70% accuracy. The results showed that ATR-FTIR spectroscopy combined with chemometrics could effectively distinguish the serum of patients with lung cancer from that of healthy people.

## Data Availability Statement

The raw data supporting the conclusions of this article will be made available by the authors, without undue reservation.

## Ethics Statement

The protocol was approved by the Ethics Committee of Yunnan Normal University (Number: ynnuethic2021-13). The patients/participants provided their written informed consent to participate in this study.

## Author Contributions

XY designed the project and completed all the research. KQ, JY, ZB, and WY provided medical instruction. XY, QO, YS, and GL wrote the manuscript. All authors contributed to the article and approved the submitted version.

## Funding

This work was supported by the National Natural Science Foundation of China (Grant No. 31760341), Yunnan province University Science and Technology Innovation Team Support Scheme.

## Conflict of Interest

The authors declare that the research was conducted in the absence of any commercial or financial relationships that could be construed as a potential conflict of interest.

## Publisher’s Note

All claims expressed in this article are solely those of the authors and do not necessarily represent those of their affiliated organizations, or those of the publisher, the editors and the reviewers. Any product that may be evaluated in this article, or claim that may be made by its manufacturer, is not guaranteed or endorsed by the publisher.
